# Design Optimization of Coronary Stent Based on Finite Element Models

**DOI:** 10.1155/2013/630243

**Published:** 2013-10-03

**Authors:** Hongxia Li, Tianshuang Qiu, Bao Zhu, Jinying Wu, Xicheng Wang

**Affiliations:** ^1^State Key Laboratory of Structural Analysis for Industrial Equipment, Department of Engineering Mechanics, Dalian University of Technology, Dalian 116024, China; ^2^Department of Electronic Engineering, Dalian University of Technology, Dalian 116024, China; ^3^Surface Engineering Laboratory, School of Materials Science and Engineering, Dalian University of Technology, Dalian 116024, China

## Abstract

This paper presents an effective optimization method using the Kriging surrogate model combing with modified rectangular grid sampling to reduce the stent dogboning effect in the expansion process. An infilling sampling criterion named expected improvement (EI) is used to balance local and global searches in the optimization iteration. Four commonly used finite element models of stent dilation were used to investigate stent dogboning rate. Thrombosis models of three typical shapes are built to test the effectiveness of optimization results. Numerical results show that two finite element models dilated by pressure applied inside the balloon are available, one of which with the artery and plaque can give an optimal stent with better expansion behavior, while the artery and plaque unincluded model is more efficient and takes a smaller amount of computation.

## 1. Introduction

Atherosclerosis is one of the most serious forms of cardiovascular disease which is one of the principal causes of mortality. Currently, three of the most common treatments for a narrowed or weakened coronary artery disease are coronary artery bypass grafting, percutaneous transluminal coronary balloon angioplasty, and percutaneous transluminal coronary stenting with the aid of coronary balloon angioplasty. Of these, the coronary stent market increased rapidly because of their high initial success rate, minimal invasive nature, and improved long-term effectiveness compared to coronary artery bypass grafting or coronary balloon angioplasty. When a stent is used, it is collapsed to a small diameter and put over a balloon-tipped tube called a catheter. With the balloon inflating the stent expands, locks in place, and forms a scaffold to hold the artery open and improve blood flow. Although stent technology has been greatly improved since its inception, many problems remain in which related to restenosis (i.e., the artery that was opened begins to become narrowed again within months of the procedure). This phenomenon is related to both arterial injury and inflammatory response of the wall against the stent struts. Therefore, efforts aiming at reducing the injury caused by stent implantations remain very meaningful. 

Previous studies indicated that the dogboning phenomenon (i.e., the ends of a stent opening first during expansion), which is due to nonuniform balloon-stent expansion, has a significant impact on thrombosis and hyperplasia development [1, 2]. This mechanical injure, that is, caused by the warped struts in the stent is often thought to induce an inflammatory response, which results in thrombosis and affects artery restenosis [3–6]. The effects of balloon length and compliance on vascular stent expansion were investigated by Cui et al. [7]. It is also believed that the stent design may affect stent expansion performance, including the dogboning phenomenon. Thus, it is important in stenting to predict and optimize the dogboning effect before manufacturing the stent. 

There are many published studies that investigated the mechanical properties of stent expansion. The mechanical properties of balloon-stent system expansion were simulated by the loading of radial displacement applied on inner surface of balloon [3, 8]. It is a simplified loading which can reduce the amount of computation but not close to the real. In addition, a time-related pressure was applied on the balloon surface to analyze the characteristics of balloon-stent system dilation [4, 5]. This loading is much close to the real situation but the interaction between the stent and the vessel wall was not considered. Moreover, multicontact including plaque/stent, balloon/stent, and plaque/balloon was considered and the radial displacement on balloon was used to dilate the balloon-stent system [6]. Clearly, this FEA model is more complete, but the loading is still simplified. Subsequently, the balloon-stent system dilation in narrowed artery was modeled with the loading mode of a time-related pressure applied on inner surface of balloon [9]. This FEA model with the internal pressure loading is much closer to the real situation, but it takes alot of computing because of nonlinearities, such as elastoplasticity, large deformation, and multicontact. In the present paper, the four common finite element models of stent expansion mentioned above are used to design optimization for reducing dogboning effect, respectively. 

The dogboning rate is an important index for assessing the quality of stent expansion [10]. It is a nonlinear, implicit function of the geometrical parameters and internal pressure loads for the stent, these are typically evaluated by the solution of the finite element method (FEM). Depending on the fidelity of simulation for stent dilation, it can become computationally expensive, limiting structural optimization of the stent. Therefore, it is challenging to reduce the computational cost of predicting the dogboning rate during the optimization process. Consequently, some approximation models are widely used during engineering to construct simplified approximations for the analysis codes, providing a surrogate model of the original code. In the present paper, we use Kriging models as alternatives to traditional second-order polynomial response surfaces for constructing global approximations for stent optimization. The Kriging model [11, 12], a semiparametric approach that does not rely on any specific model structure, is much more flexible than approaches based on parametric behavioral models [13].

In terms of stent optimization, the expansion behaviors of several stents with different given geometries were compared in terms of dogboning, foreshortening, elastic recoil, and so forth. [5]. It is easy to perform and analyze the effective factors, but the obtained “optimal stents” are only better combinations of geometry parameter levels, not the optimal solution in the design space. An adaptive optimization method based on Kriging surrogate model with a “space-filing” sampling strategy named rectangular grid is here proposed to minimize the absolute value of dogboning rate of the stent in expanding process. Kriging surrogate model can build an approximate function relationship between the stent dogboning rate and design variables (the geometrical parameters of the stent), replacing the expensive FEM reanalysis of dogboning rate in the optimization. The adaptive process is implemented by EI function. It can balance local and global search and tend to find the global optimal design. The optimization iterations are based on the surrogate model for reducing the high computational cost. 

## 2. Materials and Methods

### 2.1. Finite Element Models

**Figure 1:**
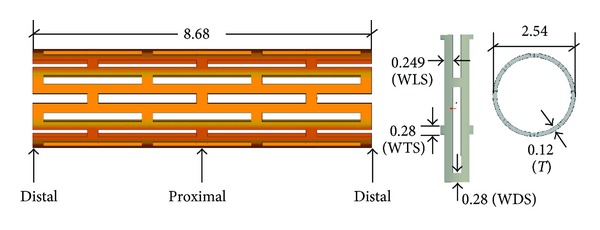
Stent model and geometric variables (mm). WLS, WTS and WDS are the width of the struts.

ANSYS finite element package was used to perform the numerical simulations. A typical diamond-shaped coronary stent (shown in Figure 1) was investigated in this study. A balloon with an 11.4 mm length and a 0.12 mm thickness was placed inside the stent. Its outside diameter was equal to the inside diameter of the stent. The stent was not in contact with the plaque at the beginning of the dilatation process. The outside surface of the plaque was adhered to the inner surface of the artery. 

Bilinear elastic-plastic, hyperelastic (Mooney-Rivlin) and linear isotropic (nearly incompressible) materials are here assumed for the slotted tube stents, balloon, and tissue (plaque and artery), respectively. All the material properties inputted are based on the data available from previous studies [2, 9].

**Figure 2:**
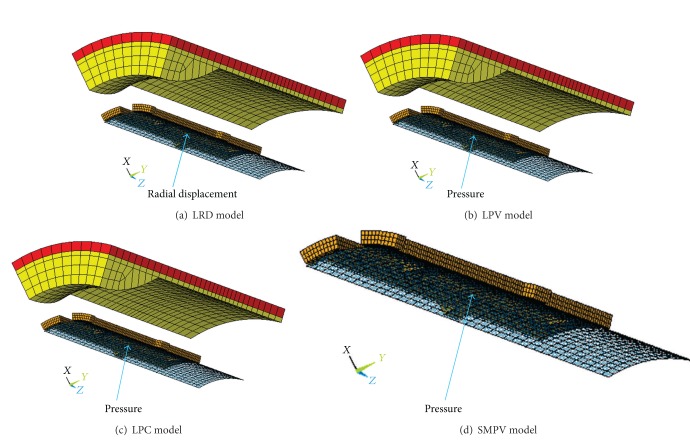
FEM models. (a) LRD model: loaded by a radial displacement to expand the diameters of stent from 2.54 mm to 4.54 mm. (b) LPV model: loaded by a pressure to expand the diameters of stent from 2.54 mm to 4.54 mm. (c) LPC model: loaded by a constant pressure. (d) SMPV model: without artery and plaque, loaded by a pressure to expand the diameters of stent from 2.54 mm to 4.54 mm.

**Figure 3:**
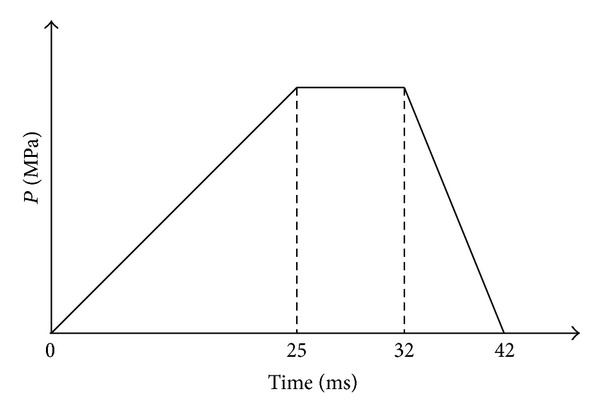
Time-related pressure.

The four FEA models are here constructed. LRD model (shown in Figure 2(a)) is loaded by a radial displacement applied on the inner surface of balloon to expand the diameters of stent from 2.54 mm to 4.54 mm. This is to allow the stenotic segment to be opened corresponding to the health artery (diameter 4.54 mm in this study). It is discretized by 11815 elements and 10180 nodes, in which the plaque, artery, stent, and balloon consist of 500 solid elements, 330 solid elements, 5103 solid elements, and 600 shell elements, respectively. The contact pairs of balloon/stent, plaque/stent, and balloon/plaque consist of 5282 contact elements. LPV model (shown in Figure 2(b)) is the same as LRD model except the loading method. This model is loaded by a time-related pressure (shown in Figure 3). It should be noted that the pressures are varied with different stent geometries. The binary-search method was used to find the pressures used to dilate the proximal region of the stents (see Figure 1) to the nominal diameter (4.54 mm in this study) after unloading. This was done to allow the stenotic segment to be opened in agreement with a health artery (diameter 4.54 mm in this study) after stent dilation. LPC model (shown in Figure 2(c)) is similar to LPV model except the constant pressure. The last one is SMPV model which has the same loading method as LPV model, but artery and plaque were not considered (shown in Figure 2(d)). It is discretized by 8004 elements and 8444 nodes, in which the stent and balloon consist of 5103 solid elements and 600 shell elements, respectively. Only one contact pair of balloon/stent consists of 2301 contact elements.

**Figure 4:**
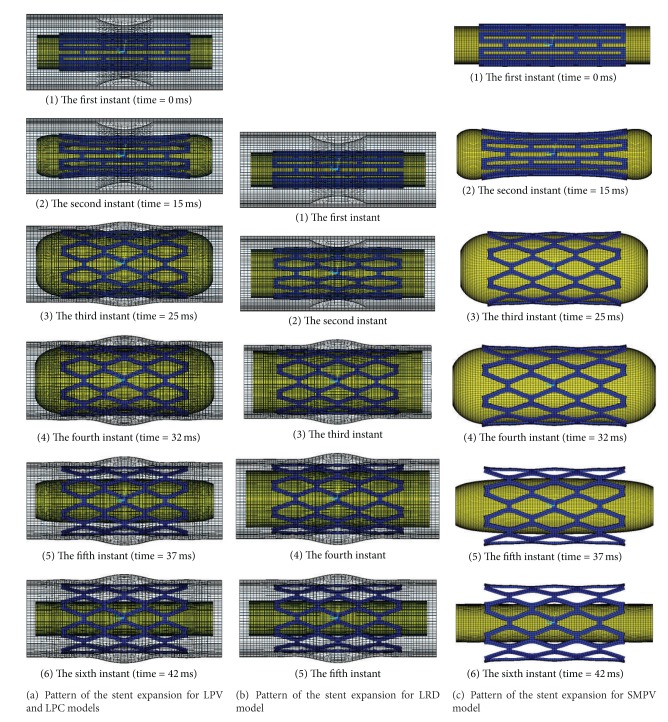
Pattern of the stent expansion based on the four FEA models.

The pattern of the transient nonuniform stent expansion based on the four FEA models is shown in Figure 4. Based on LPV, LPC, and SMPV model, the radial displacement in the distal region of the stent is larger than the proximal displacement at the second instant shown in Figures 4(a) and 4(c). However, the radial displacement in the distal region of the stent is closed to the proximal displacement since the third instant is shown in Figures 4(a) and 4(c), corresponding to the final phase of the expansion and unloading. These results are compared favorably with those reported in the literature [10, 14, 15] while based on LRD model; the radial displacement in the distal region of the stent is almost equal to the radial displacement in the proximal region of the stent because of the constant displacement loaded on the inner surface of balloon (shown in Figure 4(b)). 

### 2.2. Optimization Problem

Generally, the dogboning effect exists throughout the expanding process. It usually reaches its maximum in the beginning of loading [16, 17], but the struts are not in contact with the vessel wall. From 25 ms to 32 ms, the stent approaches an approximately cylindrical shape (corresponded to regime of the third and fourth instant appeared during the expansion of stent shown in Figures 4(a) and 4(c)), and the dogboning effect is relatively small, but stent radial displacement reaches the maximum, pushing against the artery. The dogboning observed during this period can cause serious transient mechanical injury to vessel wall. The dogboning rate of stent is here defined as

(1) $$\begin{array}{c} \text{Dogboning}\;\text{Rate}=\frac{d_{\text{radial}}^{\text{distal}}-d_{\text{radial}}^{\text{proximal}}}{d_{\text{radial}}^{\text{proximal}}}, \end{array}$$

where *d*_radial_^distal^ and *d*_radial_^proximal^ are the distal and proximal radial displacements of stent, respectively. Because the radial of stent reaches its maximum at the ending time of loading process (i.e., 32 ms), moreover, the *d*_radial_^distal^ is very large, which will induce transient mechanical damage to vessel wall, the optimization problem of the coronary stent for expanding process can be defined as follows:

(2) Min⁡ f(x)=|dradialdistal(x)−dradialproximal(x)dradialproximal(x)| S.t       x_≤x≤x¯,

where **x** is a vector of design variables, which consists of the geometrical parameters such as WDS, WTS, WLS, and *T* in Figure 1, *f*(**x**) is an objective function, and *d*_radial_^distal^(**x**) and *d*_radial_^proximal^(**x**) are the distal radial displacement and proximal radial displacement of stent at the 32 ms for LPV, LPC, and SMPV models, while for LPD model, they are the distal radial displacement and proximal radial displacement of stent after unloading. $\underline{x}$ and $\overset{-}{x}$ are lower and upper limits of the design variables (here 0.22 ≤ WDS ≤ 0.34, 0.22 ≤ WTS ≤ 0.34, 0.2 ≤ WLS ≤ 0.3, 0.1 ≤ *T* ≤ 0.14). 

### 2.3. Kriging Model

#### 2.3.1. Approximation Method

The Kriging model is described as a way of modeling a function as a realization of a stochastic process, so it is named the “stochastic process model”, which can be written as

(3) $$\begin{array}{c} \hat{y}\left(x^{i}\right)=F\left(\beta ,x^{i}\right)+z\left(x^{i}\right)=f^{T}\left(x^{i}\right)\beta +z\left(x^{i}\right) \end{array}$$

in which **x** ^*i*^ = {*x*_1_^*i*^, *x*_2_^*i*^,…, *x*_*m*_^*i*^} is the *i*th sample point with *m* variables; $\hat{y}(x^{i})$ is an approximate function fitted to *n* sample points; **f**(**x** ^*i*^) is a linear or nonlinear function of **x** ^*i*^; **β** is the regression coefficient vector to be estimated; and *z*(**x** ^*i*^) is the stochastic function, with a mean of zero and a variance *σ* ^2^. The spatial correlation function between stochastic functions is given by

(4) corr[z(xi),z(xj)]=R(θ,xi,xj)=∐l=1mexp⁡[−θ(xli−xlj)2],

where *R*(*θ*, **x** ^*i*^, **x** ^*j*^) is the Gaussian correlation function with *θ*, which characterizes the spatial correlation between two samples. Parameters can be estimated by maximizing the likelihood of samples

(5) β^=fTR−1yfTR−1fσ^2=(y−fTβ^)TR−1(y−fTβ^)nθ^=min⁡{ψ(θ)≡|R|1/nsσ2},

where **f** = [*f*_1_, *f*_2_,…, *f*_*n*_]. The estimates $\hat{\beta }$ and ${\hat{\sigma }}^{2}$ can then be obtained from (5). 

#### 2.3.2. Predictor

The function value $\hat{y}(x^{*})$ at a new point x* can be approximately estimated as a linear combination of the response values of sample **Y**:

(6) $$\begin{array}{c} \hat{y}\left(x^{*}\right)=c^{T}Y. \end{array}$$

The mean squared error (MSE) of this predictor is minimized with unbiased estimation, which gives

(7) $$\begin{array}{c} \hat{y}\left(x^{*}\right)=f\left(x^{*}\right)\hat{\beta }+r{\left(x^{*}\right)}^{T}\gamma , \end{array}$$

where

(8) $$\begin{array}{c} \gamma =R^{-1}\left(Y-F\hat{\beta }\right)\\ r\left(x^{*}\right)=\left[R\left(\theta ,x_{1},x^{*}\right),\ldots R\left(\theta ,x_{n},x^{*}\right)\right]. \end{array}$$

Thus, we can predict the function value $\hat{y}\left(x^{*}\right)$ at every new point x* by using (7).

As mentioned above, the Kriging model is an interpolation model, and the Kriging predictor is a predictor that minimizes the expected squared prediction error subject to (i) being unbiased and (ii) being a linear function of the observed response values.

#### 2.3.3. Sampling Strategy

A modified Rectangular Grid (MRG) approach was used to provide sample points for building the Kriging model. Defining the range of *m* input variables as *l*_*j*_ ≤ *x*_*j*_ ≤ *u*_*j*_, *j* = 1,…, *m*, the number of levels in the *j*th dimension is *q*_*j*_. Then, the approach is performed as follows:

(1) Contract the ranges of the variables as
  (9) $$\begin{array}{c} l_{j}\leq x_{j}\leq {\hat{u}}_{j},\;{\hat{u}}_{j}=u_{j}-\frac{1}{2}\frac{u_{j}-l_{j}}{q_{j}-1},\;j=1,\ldots ,m. \end{array}$$
(2) Perform RG sampling in the contracted space as
  (10) xj(i)=lj+kj(i)u^j−ljqj−1  , kj=0,1,…,qj−1, i=1,2,…,∏j=1mqj.
(3) Add a stochastic movement to each dimension of each sample point as
  (11) $$\begin{array}{c} \frac{\alpha_{ij}}{2}\frac{u_{j}-l_{j}}{q_{j}-1}, \end{array}$$

where *α*_*ij*_ ∈ [0,1]  is from a uniform distribution.

#### 2.3.4. Expected Improvement (EI)

The simplest way for optimization is to find the minimum of the response surface which is interpolated through the Kriging method. This way can easily lead to a local minimum, even if iterations are performed. Fortunately, an “expected improvement” function can balance local and global search. This method has been viewed as an Effective Global Optimization (EGO) [17]. The “expected improvement (EI)” method computes the extent of improvement expected to achieve if sampling at a given point. Before sampling at some point **x**, the value of *Y*(**x**) is unknown. Thus, *Y*(**x**) can be regarded as a random variable normally distributed with a mean $\hat{y}(x)$ and variance *σ* ^2^ and given by the Kriging predictor. If the current best function value is *Y*_min⁡_, then we will achieve an improvement of *I* if *Y*(**x**) = *Y*_min⁡_ − *I*. The likelihood of achieving this improvement is given by the normal density function

(12) $$\begin{array}{c} \frac{1}{\sqrt{2\pi }\;\sigma \left(x\right)}\exp\left[-\frac{{\left(Y_{\min}-I-\hat{y}\left(x\right)\right)}^{2}}{2\sigma^{2}\left(x\right)}\right]. \end{array}$$

The expected improvement is simply the expected value of the improvement found by integrating over the following density:

(13) Ε[I(x)] =∫I=0I=∞I{12π σ(x)exp⁡[−(Ymin⁡−I−y^(x))22σ2(x)]}dI.

Using integration by parts, one can show that

(14) $$\begin{array}{c} Ε\left[Ι\left(x\right)\right]=\sigma \left(x\right)\left[u\Phi \left(u\right)+\phi \left(u\right)\right], \end{array}$$

where

(15) $$\begin{array}{c} u=\frac{Y_{\min}-\hat{y}\left(x\right)}{\sigma \left(x\right)}, \end{array}$$

and where Φ and *ϕ* are the normal cumulative distribution and density functions, respectively. 

The first term of (14) is the difference between the current minimum response value *Y*_min⁡_ and the prediction $\hat{y}(x)$ at **x**, penalized by the probability of improvement. Hence, this value is large when $\hat{y}(x)$ is small. The second term is the product of the root mean squared error (RMSE) *σ*(**x**) and the normal density function *ϕ*(*u*). The normal density function value is large when *σ*(**x**) is large and $\hat{y}(x)$ is closed to *Y*_min⁡_. Thus, the expected improvement will tend to be large at a point with a predicted value smaller than *Y*_min⁡_ and/or when there is alot of uncertainty associated with the prediction.

The EI method has the following advantages: it is a balance between seeking promising areas of the design space and the uncertainty in the model and can thus allow a small DOE size; it can avoid searching the areas with large function values and reduce the computational cost; it can avoid the addition of some points close to each other in the design space that may lead to instability of the Kriging model.

#### 2.3.5. The Convergence Criterion

The convergence criterion is here to satisfy

(16) $$\begin{array}{c} \frac{\text{EI}\left(x_{k}\right)}{Y_{\max}-Y_{\min}}<\varepsilon_{1},\\ \left|\tilde{f}\left(x_{k}\right)-f\left(x_{k}\right)\right|\leq \varepsilon_{2}, \end{array}$$

where *ε*_1_ and *ε*_2_ are the convergence tolerances. *Y*_max⁡_ and *Y*_min⁡_ are the maximal and minimal function values in samples, respectively. The left-hand side of the equation is a ratio between the maximal expected improvement and the “active span” of the responses, which is also referred to as the maximal “relative EI.” $\tilde{f}(x_{k})$ is the approximate value of the objective function obtained by Kriging model in the *k*th iteration. An advantage of this convergence criterion is that the user can set the “relative” tolerances *ε*_1_and *ε*_2_ without prior consideration of the magnitudes of the problem response.

### 2.4. Optimization Algorithm

Optimization design algorithm for coronary stent based on Kriging model is described as follows.

**Step 1**

Get a set of samples with *n*_*s*_ points (each point corresponding to a group of design variables) using MRG approach, and run ANSYS program to obtain the objective function *f*(**x**_*i*_) for the sample point *i*, *i* = 1,…, *n*_*s*_. Then, select a group of the design variables corresponding with minimum *f*(**x**_*i*_) as the initial design and set *k* = 1. To be noticed is that Binary-search method was used to find the exact pressure to dilate stent at sample point *i*, *i* = 1,…, *n*_*s*_ to nominal diameter for the optimization based on LPV and SMPV models, respectively.

**Step 2**

Build an approximate function relationship $\tilde{f}(x)$ between the objective function *f*(**x**) and design variables using Kriging model based on the trial samples obtained.

**Step 3**

Minimize $\tilde{f}(x)$ to get a modified design **x** ^(*k*)^ by means of Kriging approximate model, then compute the corresponding $\tilde{f}(x^{\left(k\right)})$ by ANSYS program.

**Step 4**

Check convergence: if convergence criteria are satisfied, then x* = **x** ^(*k*)^ and stop; else add the modified design x* into the set of samples, and *k* = *k* + 1 go to Step 2. Note that the initial design will be renewed if the modified design is better than former initial design.

In this optimization problem, MRG method was used to get the sampling points. The FEM simulation can be seen as a black-box, in which a vector **x** of design variables (i.e., WTS, WDS, WLS, and *T*) is input and the corresponding response $\tilde{f}(x)$ (i.e., the absolute value of the dogboning rate) is output. Kriging surrogate model was used as alternative to traditional second-order polynomial response surfaces for constructing a global approximate relationship between the objective function *f*(**x**) and design vector **x** based on the trial samples. After the approximate relationship between the objective function and design vector was constructed, EI function is used to balance local and global search and tends to find the global optimal design. Sequential quadratic programming optimization algorithm was employed to implement the design optimization based on max EI and obtain the modified design vector **x**_*k*_. The optimization iteration started from an initial design (here is a sample corresponding with minimum *f*(**x**) in the trial samples). The procedure of building and maximizing EI continues until the stopping criterions are reached, such as the criterion described in Section 2.3.5. The optimization process stops when the Euclidean norm between real value *f*(**x** ^(*k*)^) from FEM simulation and predictive value $\tilde{f}\left(x^{\left(k\right)}\right)$ from Kriging predictor falls below a given tolerance *ε*_1_, and the Euclidean norm between current and previous iterates falls below a given tolerance *ε*_2_.

## 3. Results and Discussion

The optimization converged after 22, 8, 13, and 12 iterations based on LRD, LPV, LPC, and SMPV model, respectively. The optimization results are shown in Table 1.

LRD model is loaded by a radial displacement applied on the inner surface of balloon (shown in Figure 2(a)). This loading mode is a simplified loading which can reduce computation consume, but it does not match the real load, weakening the impact of stent geometries (WDS, WTS, WLS, and *T*) on dogboning effect. The optimization process based on LRD model may be more time-consuming. The numerical results show that the optimal WDS is smaller than WTS, which does not meet the manufacturer's design concept.

LPV model can give the most realistic simulation of stent-balloon system expansion in narrowed artery and the optimal result is reasonable. But this model contains more elements and nodes, which will complex the FEA simulation of stent dilation.

LPC model has a same deployment pressure for all sampling designs of stent in optimization process, resulting in the expansion of stent at different degree. The optimal stent based on this model is expanded to the diameter of 5.8158 mm which is far greater than the nominal diameter of health artery (4.54 mm in this study). Thus, the optimal stent based on this FEM model is not available.

SMPV model has the same loading method as LPV model, but does not contain artery and plaque. The interaction between the vessel wall and the balloon-stent system was not considered. However, SMPV model is a simplified model and the FEM simulation is much simpler.

**Figure 5:**
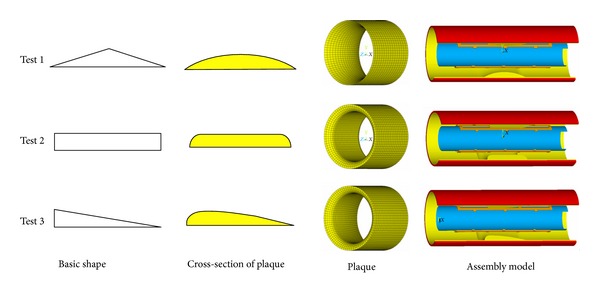
Test models with three different typical cross-section shapes of plaque. (1) Test 1: arc-shaped. (2) Test 2: bar-shaped. (3) Test 3: streamline-shaped.

Three typical plaques (shown in Figure 5) are built for the testing of optimal stents obtained based on SMPV and LPV models. The dilations of original stent and optimal stents based on SMPV and LPV models in three typical stenosed arteries are respectively simulated to get the dogboning rates of them.  Table 2 shows the results of the test, in which, the dogboning rates of stent dilation in narrowed arteries of the three typical plaques are significantly reduced, especially for the optimal stent based on LPV model. 

Based on LPV model, after optimization the dogboning rates of stent dilation in the three test models are respectively reduced by 99.93%, 85.45% and 96.17%, while based on SMPV model, those are respectively reduced by 55.72%, 50.75%, and 69.57%, as shown in Table 2. It is clearly that LPV model is more suitable for stent optimization based on FEM model. But LPV model contains more elements and nodes, which will complex the FEA simulation of stent dilation. Furthermore, Binary-search method was used to find the exact pressures to dilate the diameters of stent at sample points to nominal diameter and this will take a lot of computation. The optimal stent obtained by using SMPV model can also decrease the dogboning rates of stent dilation in narrowed artery with the three typical plaques. It is not as significantly as that obtained by using LPV model. Because WDS, WTS, and WLS of the optimal stent based on SMPV model are larger than those of the optimal stent based on LPV model, higher deployment pressures are needed to dilate stent diameters to nominal diameter in stenosed artery. When the optimal stent obtained from SMPV model is placed inside stenosed artery, stent dilation will be constrained by the raised plaque at the proximal parts of stent, so that the distal parts of stent will open first, which can cause dogboning effect. This is the reason why the dogboning effect of the optimal stent based on SMPV model dilation in stenosed arteries cannot be dismissed. But SMPV model contains fewer elements and nodes. Therefore, the corresponding FEA simulation is much simpler.

**Figure 6:**
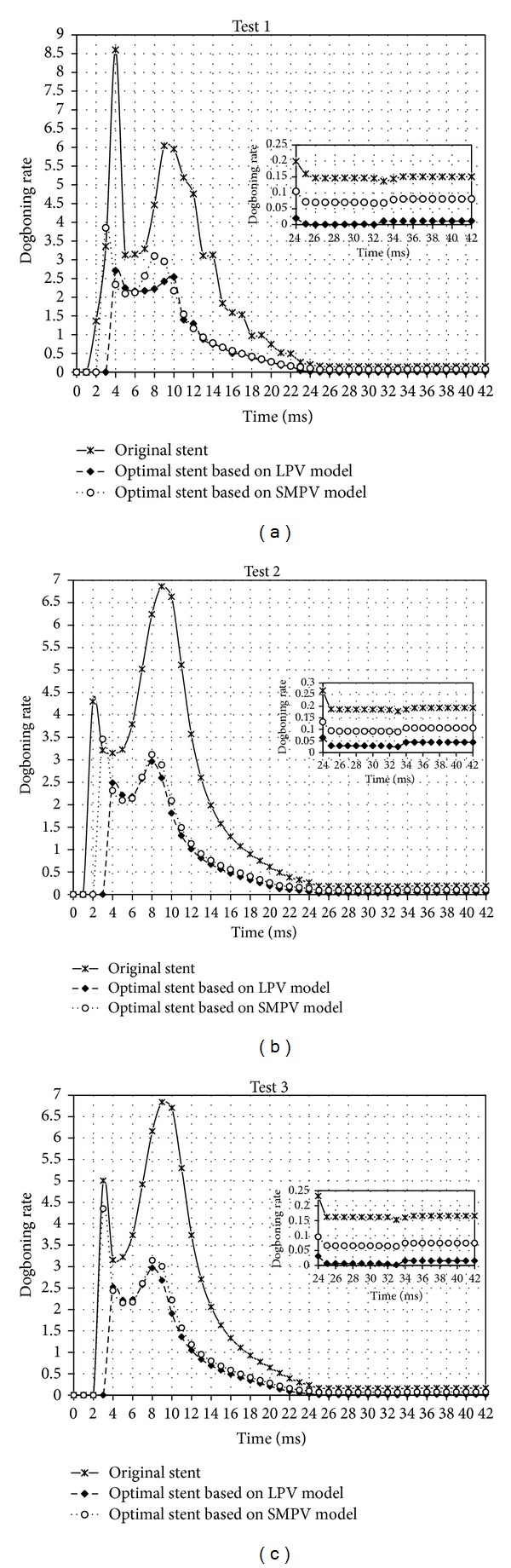
Dogboning rate for original and optimal stents along with time (mSec) of stent dilation.

The time-dogboning rate curves for original and optimal stents are shown in Figure 6 for the stent expansion process in three test models. The dogboning effect reaches the maximum at the prophase of loading stage and is reduced and remained in an almost constant value after stent expansion (corresponded to regime of the third and fourth instants, with the loading time from 25 to 32 ms, appeared during the expansion of the stent shown in Figures 4(a) and 4(c)). The radial displacement during this period reaches its maximum, and there is a strong effect of mutual contact between stent and artery wall. The dogboning observed during this period can cause serious transient mechanical injury. From the three test results, it can be seen that both the optimal stents based on LPV and SMPV models observed in the current study can reduce the dogboning significantly, especially for the optimal stent based on LPV model. 

## 4. Conclusions

In this paper, the design optimizations based on four common FEM models of stent expansion are investigated to reduce the dogboning effect by using an adaptive optimization method based on Kriging surrogate model. Plaques of three typical shapes are built for general testing of optimal stents. The results show that both LPV model and SMPV model can be used for stent optimization based on FEM model. The optimal stents based on both LPV and SMPV models can decrease dogboning effect significantly, especially for the optimal stent based on LPV model.

## Figures and Tables

**Table 1:** Optimization results.

	WDS (mm)	WTS (mm)	WLS (mm)	*T* (mm)	*P* (MPa)	Dogboning rate	Reduced by
Original stent	0.28	0.28	0.249	0.12	1.948 (LPV/LPC)	0.1452 (LPV/LPC)	—
					1.9114 (SMPV)	0.0908 (SMPV)	
					—	0.0582 (LRD)	
Optimal stent (LRD)	0.22	0.34	0.2568	0.1355	—	0.0061	89.52%
Optimal stent (LPV)	0.2367	0.22	0.2	0.1	1.7602	9.71*e* − 5	99.93%
Optimal stent (LPC)	0.2483	0.2881	0.2	0.1	1.948	0.0027	98.14%
Optimal stent (SMPV)	0.3262	0.2582	0.2056	0.1	1.7491	9.803*e* − 5	99.89%

**Table 2:** Test results.

Test model	Stent	WDS (mm)	WTS (mm)	WLS (mm)	*T* (mm)	*P* (MPa)	Dogboning rate	Reduced by
Test 1	Original stent	0.28	0.28	0.249	0.12	1.948	0.1452	—
	Optimal stent based on LPV	0.2367	0.22	0.2	0.1	1.7602	9.71*e* − 5	99.93%
	Optimal stent based on SMPV	0.3262	0.2582	0.2056	0.1	1.7950	0.0643	55.72%

Test 2	Original stent	0.28	0.28	0.249	0.12	1.9745	0.1856	—
	Optimal stent based on LPV	0.2367	0.22	0.2	0.1	1.7868	0.0270	85.45%
	Optimal stent based on SMPV	0.3262	0.2582	0.2056	0.1	1.8140	0.0914	50.75%

Test 3	Original stent	0.28	0.28	0.249	0.12	1.9555	0.1617	—
	Optimal stent based on LPV	0.2367	0.22	0.2	0.1	1.765	0.0062	96.17%
	Optimal stent based on SMPV	0.3262	0.2582	0.2056	0.1	1.7914	0.0492	69.57%
